# Deformation Behavior and Tensile Properties of the Semi-Equiaxed Microstructure in Near Alpha Titanium Alloy

**DOI:** 10.3390/ma14123380

**Published:** 2021-06-18

**Authors:** Minglang Luo, Tingyi Lin, Lei Zhou, Wei Li, Yilong Liang, Moliu Han, Yu Liang

**Affiliations:** 1 College of Materials and Metallurgy, Guizhou University, Guiyang 550025, China; minglangluo@163.com (M.L.); lintingyi2010@163.com (T.L.); zhoulei294117973@163.com (L.Z.); wli1@gzu.edu.cn (W.L.); ylliang@gzu.edu.cn (Y.L.); monewcold@163.com (M.H.); 2 Guizhou Key Laboratory for Mechanical Behavior and Microstructure of Materials, Guizhou University, Guiyang 550025, China; 3 National & Local Joint Engineering Laboratory for High-Performance Metal Structure Material and Advanced Manufacturing Technology, Guiyang 550025, China

**Keywords:** TA19 titanium alloy, semi-equiaxed microstructure, in situ SEM test, deformation behavior, mechanical properties

## Abstract

The tensile deformation and fracture behavior of a particular semi-equiaxed microstructure (S-EM) in a near alpha titanium alloy TA19 are investigated by an in situ method. In the S-EM, the thin β lamellae grow through the equiaxed α_p_ phase (α_p_), and the original α_p_/β_trans_ interface in the bimodal microstructure largely disappears, forming a blurry interface between the semi-equiaxed α_p_ phase (equiaxed α_p_ phase that is grew through by the thin β lamellae) and the transformed β microstructure (β_trans_). The formation of dense slip bands inside the semi-equiaxed α_p_ phase in the S-EM is inhibited by the thin β lamellae during the tensile deformation. The special characteristics of the S-EM reduce the stress concentration at the interface, and the crack initiation probability in the blurry semi-α_p_/β_trans_ interface decreased compared to the distinct α_p_/β_trans_ interface in a conventional equiaxed microstructure (EM). Moreover, the ultimate tensile strength of the S-EM is higher than that of the EM with a slight loss of plasticity.

## 1. Introduction

TA19 titanium alloy is a near alpha titanium alloy that is widely utilized in key structural components of aero-engines (e.g., compressor disks, blades, and casings) [1] due to its high strength to weight ratio, high temperature resistance, and corrosion resistance [2,3,4]. The mechanical properties of titanium alloys are largely influenced by the microstructural characteristics, such as the morphology and volume fraction of the α and β phases, and the grain size [5,6]. In particular, the interface characteristics between the α phase and β phase are fundamentally of significance during crack initiation and propagation [7,8,9]. For example, most of the cracks (approximately 50%) of TC21 titanium alloy (Ti-6Al-2Sn-2Zr-3Mo-1Cr-2Nb-0.1Si) generally initiate at the shear bands of the primary α lath, cracks also initiate at the α_p_/β_trans_ interface (approximately 23%) and α_s_/β interface (approximately 27%) during the tensile loading [10]. In addition, when the size of α_s_ is approximately 0.1 μm, cracks will initiate at the α_p_/β interface and α_p_/β_trans_ interface. When the size of α_s_ is approximately 0.5 μm, cracks also initiate at the α_s_/β_r_ interface [11] (α_p_: equiaxed primary α phase; α_s_: lamellar secondary α phase; β_trans_: transformed β microstructure; β_r_: remnant β phase). The phase interface in titanium alloy can effectively prevent dislocation movement and improve strength [7]. However, inconsistencies between the stress and strain easily occur at the interface during loading, resulting in stress concentration at the phase interface due to the differences in the physical, mechanical properties, and grain orientation between the α phase and β phase. Therefore, the phase interface is the preferred site for crack nucleation and propagation under tensile [10] or fatigue loading [12], which is detrimental to the mechanical properties of the alloy [13,14]. The stress concentration caused by dislocation pile-up can be reduced by refining the α phase in titanium alloy [15], but the stress concentration at the interface has not been effectively alleviated.

Different from the above-mentioned microstructural interface, the ghost α phases with blurry α_p_/β_trans_ interface characteristics in the welding heat-affected zone (HAZ) of Ti6246 alloy have been reported [16,17]. The ghost α phases are the original equiaxed α phase of the base metal, which exceeds the β phase transition temperature during welding, but the temperature and time are not sufficient to reach chemical equilibrium [17]. Xu et al. [18] also revealed that Ti6242 alloy flash welded joint obtains high ductility that is equivalent to the matrix and extra strain hardening due to the presence of the semi-equiaxed microstructure (similar to the ghost α phases) in the HAZ, but the specific effect of a pure semi-equiaxed microstructure on the mechanical properties is still unclear. Furthermore, Liang et al. [19] prepared the semi-equiaxed microstructure in TA19 titanium alloy by simulating the temperature field of the welding heat-affected zone. The tensile strength of the sample of the semi-equiaxed microstructure was 1219 MPa, which was 15% higher than that of the as-received material. However, limited efforts have been made to study the deformation and fracture characteristics of the semi-equiaxed microstructure. These studies have confirmed that the semi-equiaxed microstructure has particular microstructure and mechanical properties. Original α_p_/β_trans_ interfaces disappear in the semi-equiaxed microstructure, and whether this interface’s characteristics show special mechanical properties and deformation behavior requires further exploration.

In this study, the TA19 titanium alloy sample was rapidly heated (>100 °C/s) to β phase field (T > T_β_) and held for 35 s, thereby inhibiting the diffusion of alloying elements, and finally, a pure semi-equiaxed microstructure was prepared. An in situ tensile test was used to explore the deformation, crack initiation, and propagation characteristics of the equiaxed microstructure and semi-equiaxed microstructure. These results were combined with the results of room temperature tensile tests to further study the differences in the tensile properties caused by the two types of microstructures. The study will provide guidance for tailoring the microstructure and enriching the mechanism of the strengthening and toughening titanium alloys.

## 2. Experimental Procedures

**Figure 1:**
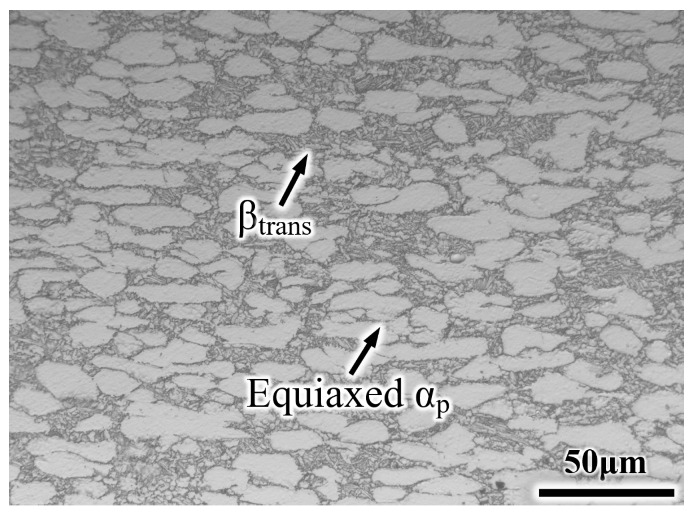
Initial microstructure of TA19 titanium alloy.

**Figure 2:**
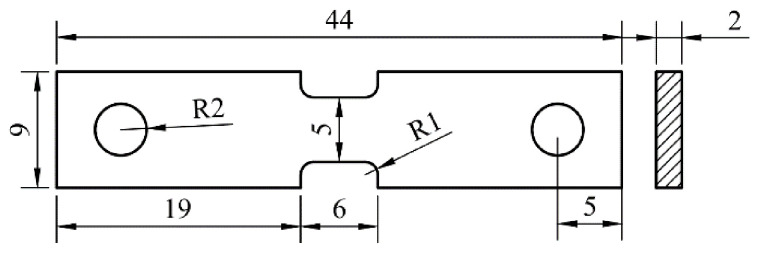
Specimen dimensions used for tensile tests.

In this work, a TA19 bar was supplied after rolling at the α + β field to obtain a plate about 65 × 35 × 170 mm^3^; then, it was tempered in the α + β field. The chemical composition of the TA19 bar is shown in Table 1. The microstructure consists of an equiaxed primary α phase (α_p_) and transformed β microstructure (β_trans_), and the transformed β microstructure contains a lamellar secondary α phase (α_s_) and remnant β phase (β_r_), as presented in Figure 1. The α/β phase transition temperature is approximately 1006 °C based on thermal analysis using a DIL805A/D phase analyzer (Netzsch, Selb, Germany). Two heat treatment modes were chosen to obtain the equiaxed microstructure (EM) and semi-equiaxed microstructure (S-EM), ensuring that the volume fraction of the equiaxed α_p_ phase, the length and thickness of the α_s_ lamellae are almost same in the two types of microstructures: (1) 970 °C/1 h, air cooling (AC) (for EM) and (2) 1015 °C/35 s, with a cooling rate of 20 °C/s (for S-EM). For optical microscopy (OM, Leica DMI5000M) (Leica, Wetzlar, Germany) and scanning electron microscopy (SEM, SUPRA40) (Carl Zeiss AG, Jena, Germany) observations, samples were ground and mechanically polished by a standard metallographic procedure (for more details, please see Ref. [20]), and they were etched with Kroll’s reagent solution (V(HF):V(HNO_3_):V(H_2_O) = 1:3:7). Moreover, microstructural parameters were counted by an Image-Pro Plus image analysis system. X-ray diffraction (XRD) analysis was carried out on a D8 Advance X-ray diffractometer with a Cu Kα source (40 kV, 200 mA) in the θ-2θ model. Electron back-scattered diffraction (EBSD) images were acquired to determine the misorientation distribution of the grains. In situ observation was performed with a SUPRA 40 scanning electron microscope at room temperature during the tensile deformation. The surfaces of the specimens were mechanically polished and strained with a crosshead speed of 0.1 μm/s. Room temperature tensile tests were carried out as standard E8/E8M Test Methods [21] on a tensile instrument (MTS 810) at a constant cross-head speed of 0.5 mm/min. Three samples with gauge dimensions of 44 × 9 × 2 mm^3^ (Figure 2) were tested to confirm the validity of the test results, and the fracture surface morphology was observed by SEM. An FEI Talos F200X field emission transmission electron microscope (TEM) (FEI, Hillsboro, OR, USA) was used to observe the microstructure after tensile fracture, thereby revealing the deformation mechanism.

## 3. Results and Discussion

### 3.1. Microstructural Characteristics

**Figure 3:**
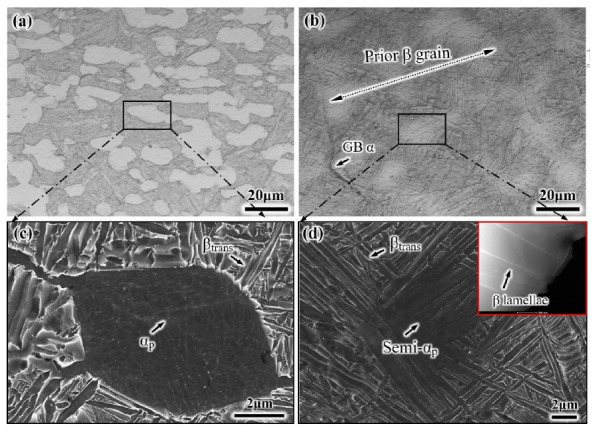
Typical microstructure after heat treatment: (**a**,**c**) EM and (**b**,**d**) S-EM.

Figure 3 shows the optical micrographs and SEM micrographs of the EM and S-EM. The morphology of the EM is almost consistent with the initial microstructure, but the volume fraction of the equiaxed α_p_ phase and α_s_ lamellae decrease (Figure 3a), which is due to allotropic transformation (α → β) after heating to the α/β phase region. The formation of the S-EM is due to the inhomogeneous diffusion of elemental Mo in the β matrix into the equiaxed α_p_ phase when the specimens are kept above the T_β_ temperature for a short time, and in the cooling process, the thin β lamellae grow through the equiaxed α_p_ phase [19]. A large amount of the equiaxed α_p_ phases remain in the S-EM because after a transient β phase treatment, only the micro-zone adjacent to the original equiaxed α_p_ phase boundary is transformed into the β phase. In this work, a relatively slow cooling rate was used to obtain the S-EM with obvious microstructural morphology, resulting in some α phases continuous precipitating along the grain boundaries (GB α) (Figure 3b). Figure 3c, d show SEM images of the EM and S-EM, respectively. Notably, there is a distinct α_p_/β_trans_ interface in the EM (Figure 3c). However, the distinct α_p_/β_trans_ interface mainly disappears in the S-EM, the thin β lamellae grow through the equiaxed α_p_ phase and exhibits a blurry interface (Figure 3d). The thickness of the thin β lamellae gradually decreases and finally disappears within the equiaxed α_p_ phase, as shown in the inset TEM image. The equiaxed α_p_ phase is grew through by the thin β lamellae in the S-EM, which is defined as the semi-equiaxed α_p_ phase (semi-α_p_) in this paper. The microstructural feature results of the EM and S-EM are presented in Table 2.

**Figure 4:**
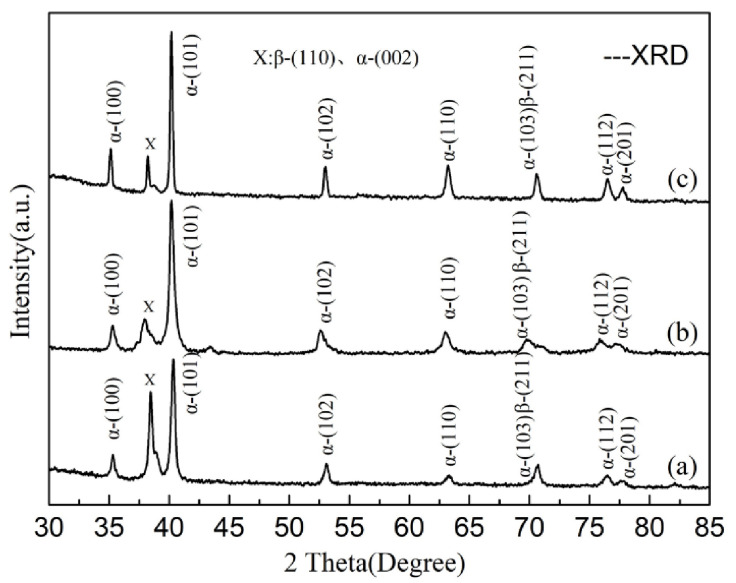
XRD patterns of the TA19 titanium alloy: (**a**) initial microstructure, (**b**) EM, and (**c**) S-EM.

Figure 4 shows the XRD patterns for the initial microstructure, the EM and S-EM of TA19 titanium alloy. The phase constitutions of the three samples are the α phase and β phase, and the intensity distribution of the diffraction peak of the α phase and β phase is similar. In the XRD patterns, α_(101)_ is the strongest among all of the diffraction peaks of both α and β phases.

**Figure 5:**
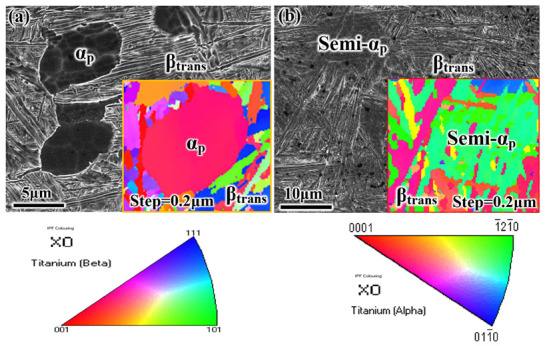
EBSD results (IPF maps) of the TA19 titanium alloy: (**a**) EM and (**b**) S-EM.

To explore the effect of the phase transition on the misorientation distribution of grains in the EM and S-EM, EBSD analysis of the microscopic regions, including the equiaxed α_p_ phase and the semi-equiaxed α_p_ phase of the two samples, were performed. The step size is 0.2 μm, as shown in Figure 5. For the EM, the equiaxed α_p_ maintains the same orientation relationship, and the fine α/β lamellae in the β_trans_ microstructure exhibit different orientation relationships (Figure 5a). For the S-EM, the semi-equiaxed α_p_ phase is formed after the phase transition of the original equiaxed α_p_ phase, which exhibits different orientation relationships. This phenomenon is different from the same orientation relationships of the equiaxed α_p_ phase in the EM. The β_trans_ microstructure in the S-EM is similar to the EM and shows multiple orientation (Figure 5b).

### 3.2. Research on the Deformation Behavior

**Figure 6:**
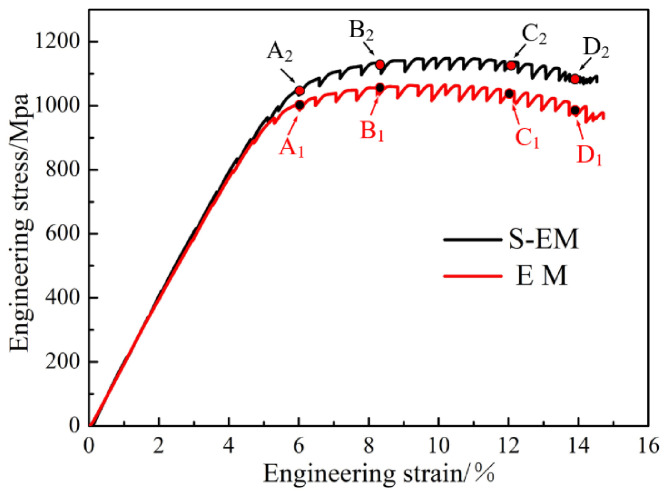
Engineering stress–strain curves for the EM and S-EM after the in situ SEM tensile test. The letters A_1_ (A_2_), B_1_ (B_2_), C_1_ (C_2_), and D_1_ (D_2_) indicate the points at which the test was interrupted for imaging.

The above description of the microstructural characteristics illustrates that the S-EM has special interface microstructural characteristics compared with the EM. An in situ tensile test is designed, as it is an effective method for comparing the deformation behavior of the two types of microstructures. During the in situ tensile process, at some strain points, the test was interrupted, and the SEM images were captured. The interval between adjacent strain points is approximately two minutes. The typical Engineering stress–strain curves of the two samples in the in situ tensile test performed at room temperature are shown in Figure 6. Moreover, the representative strain points of 6%, 8.3%, 12%, and 14% (corresponding to the letters A_1_ (A_2_), B_1_ (B_2_), C_1_ (C_2_), and D_1_ (D_2_), respectively) are selected to analyze the deformation characteristics of EM and S-EM. 

**Figure 7:**
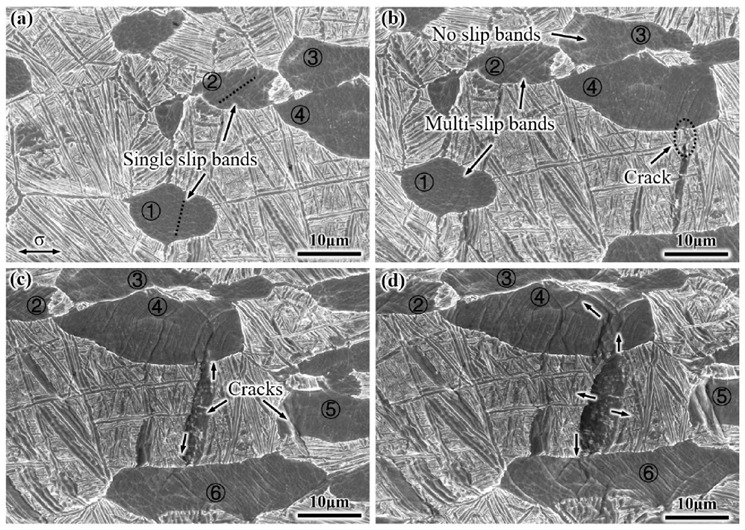
Microstructural characteristics of the EM under tensile deformation at different strain: (**a**) 6%, (**b**) 8.3%, (**c**) 12%, and (**d**) 14%. (The different equiaxed α_p_ phases in the EM are marked with ①, ②, ③, ④, ⑤ and ⑥).

**Figure 8:**
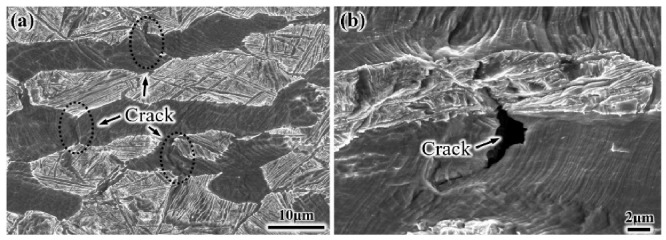
Cracks initiate at the α_p_/β_trans_ interface of the EM at different strain: (**a**) 11.6% and (**b**) 14.7%.

Figure 7 shows the actual deformation behavior of the EM in the in situ tensile test, and the tensile direction of the SEM images is parallel to the horizontal direction. When the strain is 6% (corresponds to the letter A_1_ on the curve in Figure 6), the equiaxed α_p_ phase deformation occurs first, and a small number of slip bands within the equiaxed α_p_ phase (numbers 1 and 2) are observed (Figure 7a). With a further increase in strain to 8.3% (letter B_1_), more slip bands are observed in the equiaxed α_p_ phase of numbers 1 and 2 (Figure 7b), indicating that the equiaxed α_p_ phases have good deformation capacity. However, there are no slip bands in the number 3 equiaxed α_p_ phase, and the orientation of this equiaxed α_p_ phase might not be favorable enough to initiate deformation at an early deformation stage. In addition, a large number of slip bands are hindered by the interface between the number 4 equiaxed α_p_ phase and β_trans_ microstructure, it causes local stress concentration, and a crack initiates at the α_p_/β_trans_ interface. The crack also initiates at the coarsening α_s_ lamella perpendicular to the tensile direction duo to the lamella being subjected to greater tensile stress. At a strain of 12% (letter C_1_), the crack propagates rapidly from one end along the direction of the α_s_ lamellae and from the other end along the slip band within the equiaxed α_p_ phase, forming a low-energy channel for crack propagation. The cross slip of the number 4 equiaxed α_p_ phase occurs, and the direction of crack propagation changes. Moreover, a crack also initiates in the number 5 equiaxed α_p_ phase (Figure 7c). When the strain is further increased to 14% (letter D_1_), the microstructure is severely deformed, and the length of the number 4 equiaxed α_p_ phase is increased by approximately 47% in the tensile direction. Moreover, the entire crack width is extended to 8 μm, which will lead to fracture failure of the alloy (Figure 7d). Notably, cracks also initiate at the other α_p_/β_trans_ interface when the strain is 11.6%and 14.7%, as shown in Figure 8.

**Figure 9:**
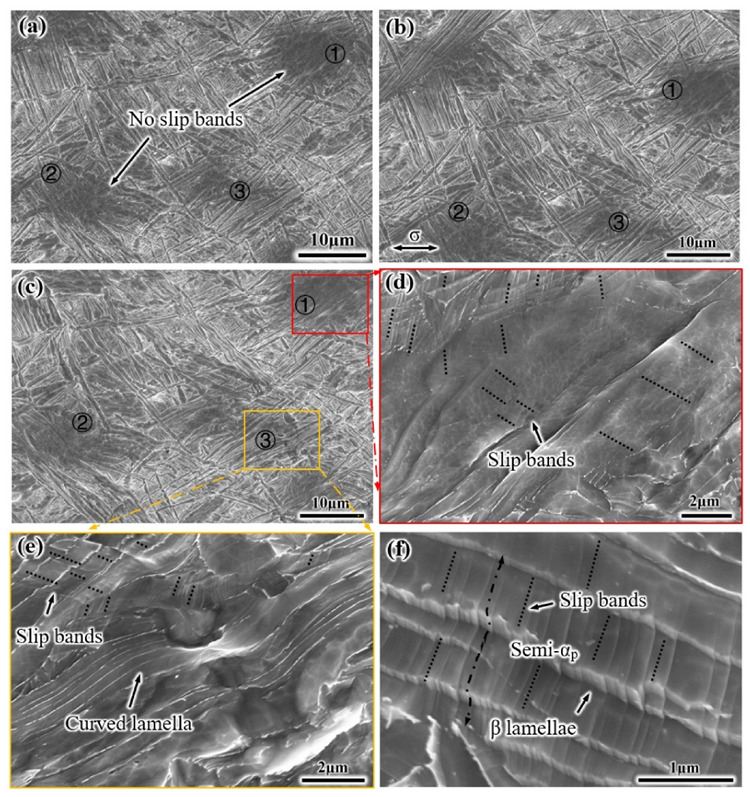
Microstructural characteristics of the S-EM under tensile deformation at different strain: (**a**) 6%, (**b**) 8.3%, and (**c**–**f**) 14%. (The different semi-equiaxed α_p_ phases in the S-EM are marked with ①, ② and ③).

For the S-EM sample, no slip bands were found in the semi-equiaxed α_p_ phase (numbers 1, 2, and 3) when the strain is 6% (letter A_2_), as shown in Figure 9a. Before the strain reaches 14% (letter D_2_), the microstructural characteristics of the S-EM have not changed significantly, so SEM images with strain of 12% (letter C_2_) are not considered. Compared to the EM, there is no obvious slip bands and cracks existing in the semi-equiaxed α_p_ phase of the S-EM when the strain is 14% (Figure 9c). The higher magnification image of Figure 9c shows that only a few and shorter slip bands occur between the thin β lamellae in the semi-equiaxed α_p_ phase (Figure 9d). These results reveal that the thin β lamellae inhibit the formation of dense slip bands in the semi-equiaxed α_p_; thereby, dislocations do not easily pile up at the blurry interface between the semi-equiaxed α_p_ phase and β_trans_ microstructure in a large amount. The bending and tearing deformation of the thin β lamellae within the semi-equiaxed α_p_ phase are observed (Figure 9e). In addition, the transfer of short slip bands across the thin β lamellae was observed in the semi-equiaxed α_p_ phase, resulting in zig-zag ledges, as shown in Figure 9f. This indicates that the thin β lamellae have a slight effect on hindering the movement of dislocations and slip transfer.

Titanium alloys exhibit many deformation behaviors, and the strain modes include planar slip, dislocation tangling, and twinning [22]. Generally, the activation of a slip system requires that a high resolved shear stress on the slip system exceeds its critical resolved shear stress (CRSS) [23]. When the resolved shear stress is higher than the critical resolved shear stress, planar slip occurs, which is the main deformation mode. Additionally, the deformation of hardly oriented grains is accompanied by dislocation tangling. During the tensile deformation, the number of slip bands and the distribution of dislocations in each grain depend on the local strain.

**Figure 10:**
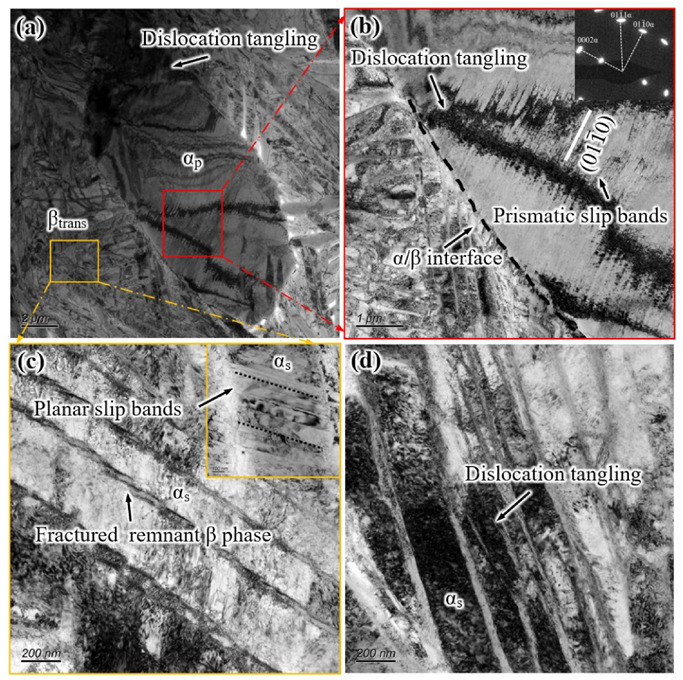
Deformation behavior of the tensile specimen with the EM: (**a**) dislocation tangling in equiaxed α_p_ phase, (**b**) prismatic slip bands in equiaxed α_p_ phase, (**c**) fractured β_r_ lamellae, and (**d**) dislocation tangling in α_s_ lamellae.

Figure 10 shows TEM micrographs of the deformation microstructure of the EM sample after tensile fracture. The observation area is approximately 1 mm away from the fracture. As shown in Figure 10a, dislocation tangling occurs at the α_p_/β_trans_ interface, which is the result of dislocation slip being hindered at the α_p_/β_trans_ interface. A large number of slip bands are arranged along a specific crystal plane, which is observed in the local higher magnification images of Figure 10a. The distance between the slip bands is approximately 70 nm. From the diffraction pattern of the selected area, these slip bands belong to the {011(-)0} prismatic slip bands (Figure 10b). Slip systems with high Schmid factor values and low CRSS (basal <a> and prismatic <a> slip) can be preferentially activated under tensile stress at room temperature, and prismatic <a> slip is more easily activated than basal <a> slip [10]. The above phenomena indicate that equiaxed α_p_ phase plays an important role in accommodating the plastic deformation of the alloy, and this result is consistent with Ref [24]. The equiaxed α_p_ phase does not maintain the Burgess orientation relationship (BOR) with the adjacent β phase, which greatly restricts slip transmission across the α_p_/β_trans_ interface [25]. So, the slip bands do not pass through the equiaxed α_p_ phase to the β_trans_ microstructure (Figure 10b), resulting in the stress concentration at the α_p_/β_trans_ interface. Meanwhile, it can be seen from Figure 10c that the remnant β_r_ phase is fractured in shear due to severe local deformation, and the short slips are observed in the coarsening α_s_ lamellae, which are also recorded as shear slip bands by Ref. [26]. Moreover, there is high density dislocation tangling in the coarsening α_s_ lamellae, which occurs because the slip motion in α_s_ lamellae is hindered at the α_s_/β_r_ interface (Figure 10d).

**Figure 11:**
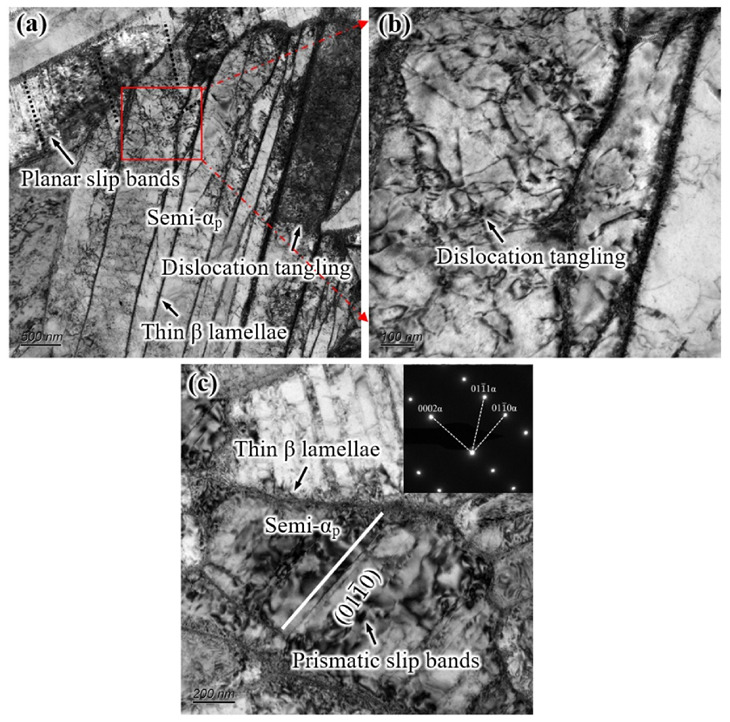
Deformation behavior of the tensile specimen with the S-EM: (**a**,**b**) dislocation tangling and planar slip bands between the thin β lamellae of the semi-equiaxed α_p_ phase and (**c**) prismatic slip bands between the thin β lamellae of the semi-equiaxed α_p_ phase.

Figure 11 shows the TEM micrographs of the deformation microstructure of the S-EM sample after tensile fracture, which is consistent with the observation area of the EM. The thickness of the thin β lamellae within the semi-equiaxed α_p_ phase in the S-EM is approximately 20 nm, which is slightly smaller than that of the remnant β_r_ phase. Different from a large number of slip bands in the equiaxed α_p_ phase of the EM, short slip bands and dislocation tangling are produced between the thin β lamellae in the semi-equiaxed α_p_ phase of the S-EM (Figure 11a); this is the result of dislocation slip being hindered by the thin β lamellae. The phenomenon of dislocation tangling between the thin β lamellae in the semi-equiaxed α_p_ phase is more obvious in the local higher magnification images (Figure 11b). The diffraction pattern shows that the slip bands between the thin β lamellae in the semi-equiaxed α_p_ belong to the {011(-)0} prismatic slip bands (Figure 11c), which is consistent with the deformation characteristics in the equiaxed α_p_ phase of the EM.

### 3.3. Tensile Properties and Fracture Characteristics

**Figure 12:**
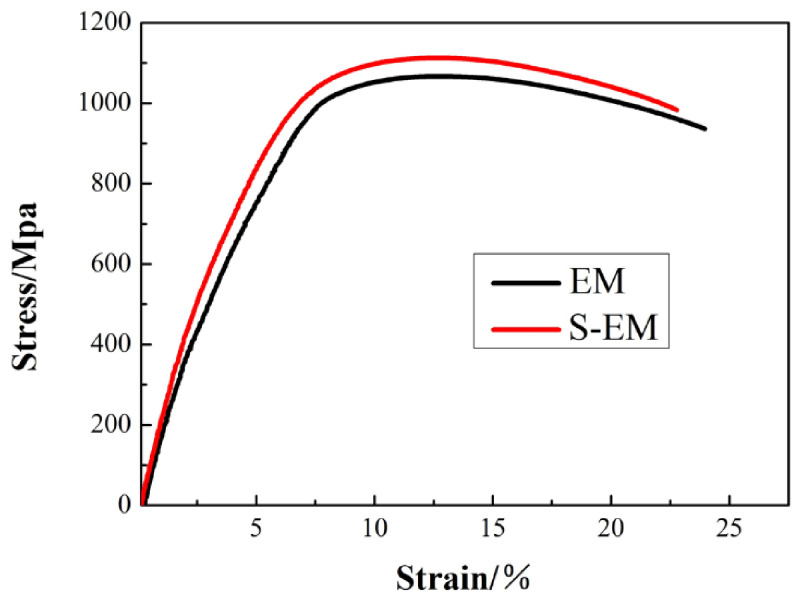
Typical engineering stress–strain curves of the EM and S-EM.

For titanium alloys, preventing dislocation slip is the most significant strengthening mechanism, in which grain boundaries and phase interfaces act as barriers to reduce the effective slip length, i.e., the Hall–Petch effect [27,28]. The typical engineering tensile stress–strain curves of the TA19 titanium alloy with the EM and S-EM at room temperature are shown in Figure 12. Furthermore, the room temperature tensile properties of EM and S-EM are summarized in Table 3. It is obvious from Figure 12 and Table 3 that the yield strength (YS) and the ultimate tensile strength (UTS) of S-EM are higher 38 MPa and 46 MPa than those of EM, respectively. On the contrary, the elongation (El) and reduction in area (RA) of S-EM are slightly lower than that of EM. The characteristics of the β_trans_ microstructure in the EM and S-EM are basically the same. Therefore, the difference in the tensile properties of EM and S-EM mainly depends on the distinct microstructural characteristics of the equiaxed α_p_ phase and semi-equiaxed α_p_ phase. The thin β lamellae grow through the equiaxed α_p_ phase in the S-EM, which hinders the slip of dislocations and reduces the effective slip length in semi-equiaxed α_p_ phase, and the strength of the S-EM is improved.

**Figure 13:**
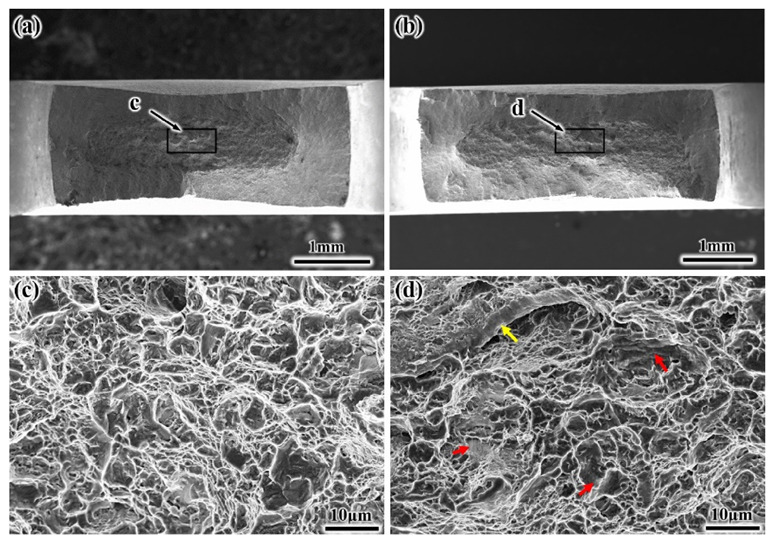
Fractographs of TA19 titanium alloy specimens with the EM and S-EM after tensile deformation showing a mixed failure mode: (**a**,**c**) EM and (**b**,**d**) S-EM.

The tensile fracture surface morphology of the EM and S-EM was analyzed to further comprehend the influence of the microstructure on the properties of the TA19 titanium alloy, as displayed in Figure 13. From the low magnification photography of the fracture surface, the shearing tip and dimple zone occur in the EM sample, and the necking characteristics are relatively obvious (Figure 13a), which is a typical feature of high plasticity. In addition, the shearing tip and dimple zone can also be observed in the macroscopic fracture surface of the S-EM, but the area of the shearing tip is slightly smaller than that of the EM, and the necking characteristic is less obvious (Figure 13b). The high magnification photography of the fracture surface shows that both the EM and S-EM tensile specimens fail in a mixed fracture mode. In the fracture surface with EM, the failure induced by dimple nucleation and coalescence along the α_p_/β_trans_ interfaces is observed, and there are tearing ridges between the dimples. The cleavage facets are relatively smooth, which is attributed to the expansion of the cracks along the equiaxed α_p_ inner slip bands or the α_p_/β_trans_ interface (Figure 13c). The dimples in the S-EM samples are small and shallow. The surfaces of the dimples are rough, and there are many small microvoids, which may be the characteristics left by the fracture of the thin β lamellae in the semi-equiaxed α_p_ phase. Furthermore, the proportion of cleavage facets (highlighted by the red arrow) increases, and traces of fracture along the grain boundary (highlighted by the yellow arrow) are found (Figure 13d).

**Figure 14:**
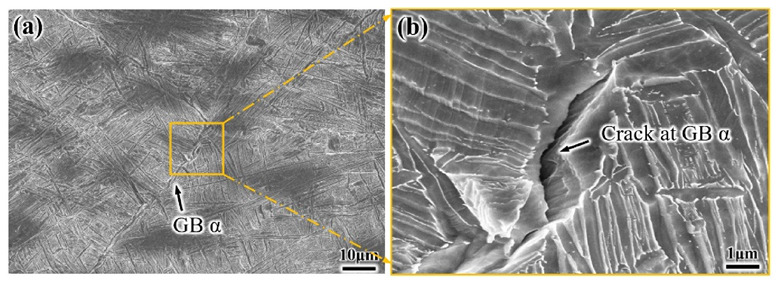
(**a**) Cracks preferentially initiate at GB α of the S-EM and (**b**) Magnified region of (**a**).

Combined with the in situ tensile test, it is found that the approximately 3 μm crack first initiates at GB α of the S-EM when the strain is 11.8% (Figure 14). In contrast, the crack first initiates at the α_p_/β_trans_ interface in the EM when the strain is 8.3%. Therefore, some GB α precipitates on the β grain boundary in the S-EM, which is an important factor that causes early fracture and ultimately leads to the decrease in plasticity [29].

**Figure 15:**
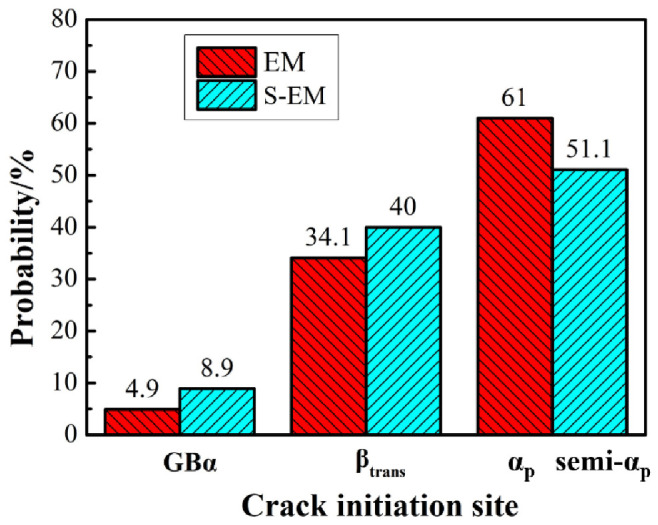
The crack nucleation probability at different sites of the EM and S-EM.

Furthermore, based on the observation and measurement of at least 100 cracks, the crack initiation probability at different sites in the EM and S-EM was obtained during in situ tensile tests, as shown in Figure 15. The probability of crack initiation in the semi-equiaxed α_p_ phase (include the blurry semi-α_p_/β_trans_ interface) of the S-EM is lower than the equiaxed α_p_ phase (include the α_p_/β_trans_ interface) of the EM, whereas the crack initiation probability increased in the β_trans_ microstructure and GB α of the S-EM in comparison to the EM. This indicates that the special interfacial microstructure of S-EM leads to the reduction of local stress concentration at the blurry interface between semi-equiaxed α_p_ phase and β_trans_ microstructure compared to the α_p_/β_trans_ interface in the EM.

**Figure 16:**
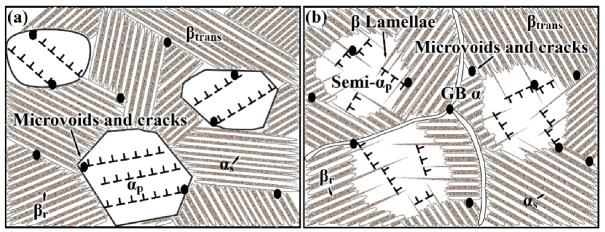
Schematic of the deformation and microvoids or cracks nucleation in different microstructures: (**a**) EM and (**b**) S-EM.

Based on the above observation and discussion of the microstructural characteristic in the EM and S-EM during the tensile deformation, the relationship between slip and the formation of microvoids or cracks is schematically illustrated in Figure 16. When the EM is subjected to a large tensile load, a large number of dislocations pile up at the α_p_/β_trans_ interface, resulting in a severe stress concentration at the α_p_/β_trans_ interface. Therefore, the α_p_/β_trans_ interface is the preferred initiation site for microvoids or cracks [10,30]. Due to the different orientations and short effective slip distances in the α_s_ lamellae and remnant β_r_ phase, the deformation capacity is relatively poor, resulting in cracks initiating at the α_s_/β_r_ interface [31], as shown in Figure 16a. For the S-EM, dense slip bands in the semi-equiaxed α_p_ phase are suppressed by the thin β lamellae, and fewer dislocations pile up at the blurry interface between the semi-equiaxed α_p_ phase and β_trans_ microstructure, which reduces the stress concentration. Then, the probability of crack initiation at the blurry semi-α_p_/β_trans_ interface decreases (Figure 16b).

## 4. Conclusions

In this work, the deformation behavior and fracture mechanism of the particular S-EM were revealed by in situ tensile tests, and the influence of the microstructural characteristics on the mechanical properties was explored. The main conclusions are summarized as follows:

(1) In the S-EM, the distinct α_p_/β_trans_ interface in the bimodal microstructure primarily disappears, and the thin β lamellae grow through the equiaxed α_p_ phase, leading to partial division of the equiaxed α_p_ phase by the thin β lamellae.
(2) The S-EM effectively suppress the formation of dense slip bands in the semi-equiaxed α_p_ phase, so the stress concentration at the blurry semi-α_p_/β_trans_ interface in the S-EM has been reduced, and the semi-equiaxed α_p_ phase and β_trans_ microstructure have better deformation compatibility. In addition, the tensile strength of the S-EM is higher than that of the EM due to the thin β lamellae hindering the movement of dislocations within the semi-equiaxed α_p_ phase in the S-EM.
(3) In the S-EM, the prismatic slip and dislocation tangling between the thin β lamellae are the main deformation modes of the semi-equiaxed α_p_ phase. The deformation of the β_trans_ microstructure in the S-EM is mainly affected by planar slip and dislocation tangling in the α_s_ lamellae.
(4) The tensile fracture failures of both EM and S-EM in TA19 alloy show a mixture fracture mode. Compared to the EM, S-EM samples have shallower dimples and more cleavage facets, which leads to the proportion of brittle fracture mechanism of S-EM is slightly larger than EM.

## Figures and Tables

**Table 1:** Chemical components of the material (wt %).

Al	Sn	Zr	Mo	W	Si	Ti
6.61	1.75	4.12	1.91	0.32	0.10	Bal.

**Table 2:** Summary of the constituent phase parameters for the EM and S-EM.

Microstructure	α_p_ (vol %)	α_p_ Diameter (μm)	α_s_ Length (μm)	α_s_ Width (nm)	GB α (vol %)	GB α Width (nm)
EM	42 ± 2	12 ± 1	10 ± 1	400 ± 20	0.3	420 ± 30
S-EM	41 ± 2	13 ± 1	11 ± 1	410 ± 20	1.2	850 ± 30

**Table 3:** Tensile properties of EM and S-EM at room temperature.

Microstructure	YS/Mpa	UTS/Mpa	El/%	RA/%
EM	984	1067	23.9	32.9
S-EM	1022	1113	22.8	30.8

## Data Availability

Not applicable.
